# Extensive Internet Involvement—Addiction or Emerging Lifestyle?

**DOI:** 10.3390/ijerph8124488

**Published:** 2011-12-02

**Authors:** Karin Helmersson Bergmark, Anders Bergmark, Olle Findahl

**Affiliations:** 1 Department of Sociology & Addiction Research Group, Stockholm University, S-10691 Stockholm, Sweden; 2 Department of Social Work & Addiction Research Group, Stockholm University, S-10691 Stockholm, Sweden; Email: anders.bergmark@socarb.su.se; 3 World Internet Institute, P.O. Box 975, S-80133 Gavle, Sweden; Email: olle.findahl@wii.se

**Keywords:** Internet, addiction, behavioral addiction

## Abstract

In the discussions for the future DSM-5, the Substance-Related Disorders Work Group has been addressing “addiction-like” behavioral disorders such as “Internet addiction” to possibly be considered as potential additions for the diagnostic system. Most research aiming to specify and define the concept of Internet addiction (or: Excessive/Compulsive/Problematic Internet Use—PIU), takes its point of departure in conventional terminology for addiction, based in established DSM indicators. Still, it is obvious that the divide between characteristics of addiction and dimensions of new lifestyles built on technological progress is problematic and far from unambiguous. Some of these research areas are developing from the neurobiological doctrine of addiction as not being tied to specific substances. The concept of “behavioral addictions”, based on biological mechanisms such as the reward systems of the brain, has been launched. The problems connected to this development are in this study discussed and reflected with data from a Swedish survey on Internet use (n = 1,147). Most Swedes (85%) do use the Internet to some degree. The prevalence of excessive use parallels other similar countries. Respondents in our study spend (mean value) 9.8 hours per week online at home, only 5 percent spend more than 30 hours per week. There are both positive and negative social effects at hand. Many respondents have more social contacts due to the use of Internet, but there is a decline in face-to-face contacts. About 40% of the respondents indicate some experience of at least one problem related to Internet use, but only 1.8% marked the presence of all problems addressed. Most significant predictors for problem indicators, except for age, relate to “time” and time consuming activities such as gaming, other activities online or computer skills.

## 1. Introduction

### 1.1. Background

The concepts “addiction” and “dependence” were originally developed and elaborated to handle the diagnosis of individuals displaying excessive and problematic use of alcohol and other drugs (e.g., opiates). Presently the two concepts are often used interchangeably [1]. Indicators for the concept include tolerance, withdrawal, preoccupation, craving, impaired control and continued use despite clear evidence of adverse consequences. Lately these indicators have been applied to not only the intake of substances but also to different behaviours and practices. In the discussions for the future DSM 5, the Substance-Related Disorders Work Group has been addressing “addiction-like” behavioural disorders such as “Internet addiction” to possibly be considered as potential additions for the diagnostic system. An expansion of the addiction concept is also advocated from different corners of the research community (see e.g., [2]).

Most research aiming to specify and define the concepts of Internet addiction (or excessive, compulsive or pathological Internet use), takes its point of departure in conventional terminology for addiction that is based in established DSM indicators. Still, it is obvious that the divide between characteristics of addiction and dimensions of new lifestyles built on technological progress is problematic and far from unambiguous. Some of these research areas are developing from the neo-biological doctrine of addiction as not being tied to specific substances. The concept of “behavioural addictions”, based on biological mechanisms such as the reward systems of the brain, has been launched. In this paper the notion of Internet addiction is discussed and illuminated with data from a Swedish general population survey on Internet use. In comparison to studies that have made use of self-selected convenience samples, such an approach opens up for a broader discussion on the prevalence and characteristics of Internet addiction (or problems related to extensive Internet involvement). 

We expected to be able to provide some indications of how experiences of Internet use problems are related to such variables as age, specific Internet activities, general computer skills and time online. Besides the inclusion of more conventional background factors (as age, sex, educational level and living conditions), we chose to include possible predictors for Internet use problems. We assumed that some specific Internet activities (e.g., gaming), general computer skills and time online would display positive correlations to problem indicators. In the case of computer skills we assumed that this reflects a general technological interest that enables the individual to use the Internet in more elaborated ways and in this way might be related to the prevalence of problems. 

### 1.2. Swedes and Internet

The significance of the Internet has gone from being a work-related tool for certain groups to something most Swedes use for many activities [3]; from gaming to bank transactions and romantic interactions. Internet gives many opportunities for interaction between individuals, without any limitations set by geography or social ties. Gaming and other social activities form a large part of many Swedes’ life online. In addition, aspects of our social life and relations have shifted—or expanded—through the upsurge of digital arenas. During the last ten years the share of Swedes who go online on a regular day has risen from 21% to 65% [4]. In 2010, 85% of the population had access to Internet in their homes [5].

### 1.3. Internet Addiction

During the last decade different forms of Internet use have been in focus for substantial research efforts. Some studies include positive aspects as well as negative. Shen and Williams [6] analysed survey and game-based data for over 5,000 EverQuest gamers and conclude that such games can have both positive and negative consequences for sociability and family life. Pre-existing social ties were reinforced via online contacts. The game also offered an alternative possibility for family communication. Gaming together in the family was positive in terms of solidifying family relations. On the other hand, gamers who did not play with family members displayed, in comparison, higher levels of loneliness. Purposes for gaming, contexts and individual features of the gamer, delineates according to these authors consequences of extended life online—as time displacement or social augmentation. 

There has also been an upsurge of problem-oriented studies that advocate concepts such as “behavioural addiction”. While neurobiological scholars advocating the concept of addiction previously used to limit discussions to “drug and alcohol addiction” (e.g., [7]), it is at present increasingly argued that many behaviours are as apt to produce short term reward, and provoke continued behaviour and diminished control [8]. The focal point is an attraction, residing in the reward system of the brain, that makes it impossible to resist an impulse to use or do a behaviour of some type. An aggravating circumstance is that comorbidity (behavioural and substance addictions) is claimed to be common [8,9,10,11]. This development has also opened the door for a widening of the boundaries concerning which activities that can be considered plausible to develop into an addiction. Some authors argue that food [12] can act as a trigger to addiction in ways similar to alcohol or tobacco. Excessive exercise has been described in similar ways [13,14]. References to pathological gambling are more common [15]. Pathological gambling is included in DSM lV and is diagnosed via indicators for tolerance, withdrawal, preoccupation, a strong desire, impaired control and continued use despite adverse consequences. 

In research from the field of “Internet addiction”, one key reference is Kimberly Young [10] and her study from the 1990s [16,17]. Drawing from DSM definitions for pathological gambling and her own empirical studies she participated in the development of the concept “Internet addiction” and in forming an instrument for assessment. In a more recent paper, Young claims that Internet based gaming constitutes a now rapidly growing form of Internet addiction, especially among children and teenagers [18]. The indicators Young uses measure salience, mood modification, tolerance, withdrawal, conflict and relapse (see also [19]) and are parcelled in eight questions.

Respondents who answered yes to five or more of the questions were classified as addicted [16]. Questions referred to all sorts of online activity. The study group was defined by self-selection from different sources; via newspaper advertisements, flyers on college campuses, Internet addiction support groups, and posted online for web-searches on “Internet addiction”. Six out of ten respondents were women (middle-aged; older than male respondents). Four out of ten had no vocational background. The support groups included “the Internet Addiction Support Group” and “Webaholics Support Group”. When the 12-step language, programme and ideology is established in the group from where respondents are found, it should not be surprising if respondents spontaneously present narratives on e.g., withdrawal, loss of control and craving. Still, Young concludes that she could distinguish a group of addicts who spent long time online “for pleasure” (mean time 38.5 hours per week), who preferred interactive sites more than others and who describe themselves as “completely hooked”. She claims that the addicts reported lots of life problems but there are no records of how this was measured. 

Gilbert *et al.* [9] used the Internet addiction test suggested by Young in a study based on a convenience sample of Second Life-users. Van Rooij *et al.* [20] developed a similar list of questions from a manual for CIU (Compulsive Internet Use). Shapira *et al*. [21] present a general definition of Internet addiction based on one symptom: loss of control and two “signs”: distress and impairment of daily routines. 

Weinstein and Lejoyeaux [10] suggest four components for the diagnosis of Internet addiction (dependence). Excessive Internet use and loss of sense of time or basic drives is the first. The second is based on indications of withdrawal; tension or depression when unable to be online. The third component is tolerance; e.g., need for more time online and the forth include adverse consequences such as arguments and social isolation. These authors are, however, quite critical of previous use of diagnostic tools for Internet Addiction and the main conclusion is that “more research is needed”.

Given the fact that there are several rather obvious problems connected to the concept of “Internet addiction” it is somewhat surprising that the Substance-Related Disorders working Group for the upcoming DSM 5 has identified Internet addiction as the main candidate for future inclusion under the new section “Addiction and Related Disorders”. O’Brien (p. 2, [22]) notes that “Other non-pharmacological addictions were also reviewed, but only gambling met the criteria for inclusion at this time: Internet addiction will be recommended for the Appendix in order to encourage further research”. A major problem related to the ambition to substantiate and delimit Internet addiction is the fact that the Internet is a technology that enables individuals to engage in an almost endless number of activities, but it is not an activity in itself. To the extent that anyone is considered to be addicted to his or her activities that are made possible through the use of the Internet, it is most likely to be activities such as online gaming, gambling, chatting or network communicating (as e.g., Facebook). 

Thus, it can be claimed that the concept of internet addiction lacks specificity. Sussman *et al*. [23] have suggested that “addiction specificity”, *i.e.*, why one pattern of addictive behaviours may be acquired whereas another is not, can be illuminated within a model that identifies four dimensions; pragmatics, attraction; communication and expectation (PACE). The first component, pragmatics, concerns the accessibility of a particular addictive behaviour. There must be a supply of the object of addiction, in the case at hand the Internet must be established in order to supply Internet gaming, gambling or chatting. An individual also needs to be aware of this supply of activities as well as have the acquisition skills of how to use computers and connect to the Internet. Lastly the individual needs to have the means of exchange in order to get access to the object of addiction (*i.e.*, economic resources that allows the individual to have an Internet connection). In the perspective of the PACE model of addiction specificity, the idea of Internet addiction leaves out the aspects of attraction, communication and expectation. The idea of Internet addiction lacks a specification of the actual activity that is the object of attraction (as e.g., gaming, gambling or chatting) and consequently the characteristics of the remaining dimensions of the PACE model—communication and expectation—cannot be identified. 

Moreover, the activities that are possible to engage in do not constitute a stable configuration; on the contrary, there is a constant transformation of activities that are provided through the Internet. An attempt to solve this type of problem is to use a terminology that has a more limited scope, e.g., the specified category of “gaming addiction”. Although such a change represents a step in the right direction it does not solve all problems. There are many different types of games and different way of playing them. Shen and Williams (p. 125, [6]) identifies “a universe of playable titles now over 30,000 dozens of genres, and myriad play options within most titles”.

## 2. Methodology

### 2.1. This Study

Research on Internet and virtual worlds is currently at hand in many disciplines. Most often this research is based on ethnographic data or surveys based on self-selected study groups [24]. We searched for more representative data where indicators used by Young [16] were included. Therefore we have, for this study, chosen to use a survey from 2009; “Swedes and Internet” [25], the Swedish contribution to the World Internet Project (WIP). In Sweden these surveys on Internet use have, in the form of a revolving panel study, been carried through almost annually since the Year 2000. Respondents are interviewed via telephone or the web (respondents’ choice). The survey from 2009 was selected since this survey included five indicators related to “Internet Addiction”. Internal dropout was for most variables not higher than 5%. Listwise deletion was used for analyses. Only Internet users (83.2% of all respondents) were included in this study; n = 1,147. The group of Internet users demonstrated similar distributions on background variables as the whole group of respondents. There is no information available on external drop-outs and, for a survey with this kind of design, drop-out rates are difficult to calculate. We would expect overrepresentation of respondents with an interest for computers and Internet. On the other hand, the frequency of Internet users in this study parallels other sources for national Internet statistics [26]. Also, background characteristics are comparable to other national Internet data. 

### 2.2. Methodology and Analyses

For this study we included a number of independent variables. Demographics included sex (gender), year of birth and residence (urban/rural). Educational level was measured with highest degree obtained. For occupation we constructed an indicator with two positions (1: working or student; 2: unemployed, on parental leave, on sick leave, or on pension leave). An indicator of household income was deleted due to high missing data on that question (*i.e.*, 35%). 

Gaming experiences were assessed with the item “How often, if ever, do you use Internet for gaming” (never–several times per day) and gambling experiences was assessed with “How often, if ever, do you use Internet for gambling” (never–several times per day). Membership in communities on Internet (e.g., Facebook) as well as other kinds of online activities were assessed. Computer skills was assessed with the items, “How skilled do you consider yourself to be when it comes to computer use?” (not at all skilled–very skilled) and “Do you know how to install an operating system on a computer?” (no–yes). Indicators of social life was assessed with the items, “Has the use of Internet led to less/the same/more contact with your family/friends?” (much less–much more) and “Would you say that since you started to use the Internet you spend less/the same/more time in face-to-face contact with your family/friends?” (less time–more time).

As a measure for time online we used a question on how much time (in hours and minutes) during a regular week the respondent spends on Internet at home. This does not directly correspond to Young’s data on time “for pleasure” online but is the closest we can get though not including online activities at work or in school. 

As indicators of Internet related problems we included all five indicators available. These were: “Have you ever spent too much time on Internet”, “Do you ever come to quarrel with your family over (your) excessive Internet use”, “Do you ever feel depressed, irritated or annoyed because you cannot be online”, “Did you ever forget about usual needs such as eating or sleeping due to Internet”, and “Have you ever tried and failed to cut down on time on Internet?” All five indicators include four response alternatives: never, sometimes, often, very often. For the analysis the indicators were, however, recoded into occurrence (no/yes) due to the imprecision of the alternative answers and resulting skewness of data distributions (*i.e.*, the distance between “sometimes” and “often” is not necessarily the same as between “often” and “very often”). 

Linear regression models were used to explore predictors of Internet related problems. For the first analysis, occurrence of the five problem indicators was summed. The constructed dependent variable was skewed and therefore transformed to logarithmic form for the analysis. Following this, a similar regression model using “time on Internet”, also in logarithmic form due to skewness, was carried through. Both regression models were tested for collinearity, with satisfactory results (see Tables).

## 3. Results

### 3.1. Sample Characteristics

In Table 1, background variables for the study group are presented. Half the group was female and a majority (64.8%) lived in urban settings. The oldest respondent was born in 1917 and the youngest in 1996, with a median and mean age of 45. Approximately, twenty-five percent held a university degree while approximately 20% reported only obtaining elementary school education. A majority of respondents (64.1%) were working. 

**Table 1:** Respondents.

**Gender male/female**	50.4%/49.6%
**Age**	Mean = 45 Md = 45
**Urban/rural residence**	64.8%/35.2%
**Educational level**	22.3% Elementary school exam only
	55.5% Secondary level
	27.2% University degree
**Occupation**	64.1% Work
	12.0% Students
	13.1% Retired
	10.8% Unemployed, sick- or parental leave

**Figure 1:**
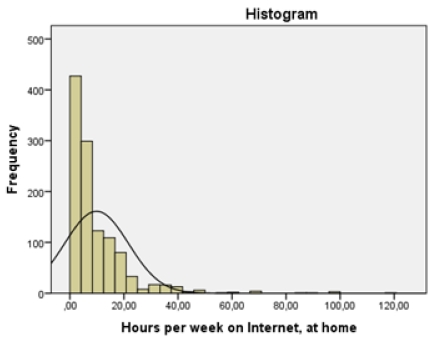
Hours per week on Internet, at home (Mean = 9.86, Std. Dev = 11.823, N = 1,147).

Among Internet users, the mean time length on Internet, at home, was 9.8 hours per week (median= 7 hours; see Table 2). The distribution is heavily skewed (see Figure 1). One out of three used the Internet for gaming while it was quite unusual to use Internet for gambling (11.4%). Internet was also used for many other kinds of activities. None of these other activities that were addressed (*i.e.*, instant messaging, chatting, unspecified Internet surfing, surfing sex sites, posting photos) correlated significantly with the problem indicators used for analyses when introduced in regression models and they were not included in further analyses. Approximately 75% considered themselves as having good or even very good skills in computer use and 45% reported that it was easy or very easy to solve computer related problems and claimed to know how to install a new operating system in a computer.

**Table 2:** Internet use and computer skills among Internet users.

**Hours spent on Internet at home a regular week**	9.8 (mean value) 7.0 (median value)
**Hours spent on Internet (all locations) a regular week (includes school/work)**	15.6 (mean value)
**Internet gaming; ever**	33.7%
**Hours spent on gaming a regular week among regular Internet gamers**	6.1 (mean value)
**Internet gambling; ever**	11.4%
**Instant messaging; ever**	48.5%
**Internet chatting; ever**	16.3%
**Internet surfing (unspecified); ever**	75.2%
**Internet sex sites; ever**	18.3%
**Posting photos on Internet; ever**	33.8%
**Skills in computer use; good or very good**	73.3%
**Knows how to install a new operating system**	45.0%
**Member of Internet based community**	29.4%

Almost one out of three respondents was member in at least one community on the Internet. More said (see Table 3) that use of Internet had led to more contact, both with family members and with friends, than the opposite. More respondents endorsed, however, the fact that Internet use had led to less face-to-face contact than the opposite, especially with other family members.

**Table 3:** Internet and social life among Internet users.

**Has the use of Internet led to less/more contact with family members?**	5.5% less 28.5% more
**Has the use of Internet led to less/more contact with friends?**	4.3% less 39.9% more
**Have you spent less/more time in face-to-face interaction with your family since you got Internet at home?**	27.5% less 3.2% more
**Have you spent less/more time in face-to-face interaction with your friends since you started to use Internet?**	9.8% less 3.9% more

### 3.2. Regression Analyses

In the first regression analysis (Table 4), the summed occurrence of problem indicators was tested against background variables and Internet related variables. Among the background variables only (young) age resulted in a significant prediction. The most powerful predictor was prevalence of membership in online communities, followed by time on Internet and prevalence of gaming. Also, prevalence of gambling predicted more problems. More skills in computer handling were also significant predictors, as were spending less time face-to-face with family and friends since the Internet was introduced in the home, while degree of contact with friends (or family) did not predict problems. 

**Table 4:** Linear regression analysis; (ln) summed occurrence of problem indicators.

Model		Unstandardized Coefficients		Standardized Coefficients	t	Sig.	Collinearity Statistics	
		B	Std. Error	Beta			Tolerance	VIF
	(Constant)	-4.851	2.259		-2.147	0.032		
	Woman	0.010	0.030	0.009	0.330	0.741	0.785	1.274
	Year of birth (young age)	0.003	0.001	0.080	2.345	0.019	0.554	1.806
	Educational level	0.010	0.011	0.025	0.918	0.359	0.889	1.125
	Rural living	-0.008	0.029	-0.007	-0.268	0.788	0.945	1.058
	No engagement (no work/stud.)	0.030	0.037	0.024	0.789	0.431	0.717	1.394
	Internet gaming	0.056	0.012	0.135	4.724	0.000	0.784	1.276
	Internet gambling	0.077	0.022	0.095	3.544	0.000	0.899	1.112
	Degree of skills related to computer use	0.069	0.023	0.093	3.051	0.002	0.687	1.456
	Knows how to install operative systems	0.109	0.034	0.103	3.243	0.001	0.637	1.571
	Member in community/-ies	0.199	0.035	0.172	5.703	0.000	0.704	1.421
	Less or more FTF with friends	-0.154	0.040	-0.106	-3.819	0.000	0.834	1.200
	Less or more FTF with family	-0.065	0.030	-0.061	-2.176	0.030	0.822	1.216
	Less or more contact with family	-0.043	0.025	-0.065	-1.763	0.078	0.470	2.126
	Less or more contact with friends	0.014	0.023	0.023	0.598	0.550	0.446	2.240
	Hours/week on Internet, at home	0.000	0.000	0.149	5.096	0.000	0.750	1.333

Dependent Variable: ln problem indicators in dummy form and summed. Adj. R² = 0.272.

In the second regression analysis (Table 5), time in hours per week on Internet at home was tested. Young age and unemployment (no work/studies) predicted more time on Internet, which might be as expected. Gaming constituted the most powerful predictor, but skills with computers and membership in Internet based communities also predicted more time spent on the Internet. Stating that the Internet has led to more contact with friends predicted more time on Internet while there was no significant prediction value in changes of face-to-face contacts.

**Table 5:** Linear regression analysis; (ln) summed hours per week on Internet, at home.

Model		Unstandardized Coefficients		Standardized Coefficients	t	Sig.	Collinearity Statistics	
		B	Std. Error	Beta			Tolerance	VIF
	(Constant)	-12.650	5.282		-2.395	0.017		
	Woman	-0.115	0.071	-0.046	-1.620	0.106	0.785	1.273
	Year of birth (young age)	0.008	0.003	0.100	2.998	0.003	0.558	1.792
	Educational level	0.005	0.026	0.005	0.179	0.858	0.890	1.124
	Rural living	-0.104	0.067	-0.039	-1.542	0.123	0.948	1.055
	No engagement (no work/stud.)	0.351	0.087	0.117	4.021	0.000	0.728	1.373
	Internet gaming	0.189	0.027	0.192	7.046	0.000	0.838	1.193
	Internet gambling	0.057	0.051	0.029	1.123	0.262	0.901	1.109
	Degree of skills related to computer use	0.242	0.053	0.138	4.615	0.000	0.697	1.434
	Knows how to install operative systems	0.413	0.078	0.163	5.265	0.000	0.649	1.542
	Member in community/-ies	0.235	0.082	0.085	2.864	0.004	0.706	1.417
	Less or more FTF with friends	0.110	0.095	0.031	1.156	0.248	0.834	1.199
	Less or more FTF with family	-0.005	0.070	-0.002	-0.075	0.940	0.823	1.215
	Less or more contact with family	0.093	0.058	0.058	1.609	0.108	0.472	2.117
	Less or more contact with friends	0.282	0.054	0.194	5.227	0.000	0.450	2.223

Dependent Variable: ln hours per week on Internet, at home, Adj. R² = 0.296.

## 4. Conclusions and Discussion

Much of the previous research in the field of Internet and other behavioral addictions is based on selected samples; in the case of Internet addiction, the research has been biased towards high frequency users of Internet. In the present study this is not the case. As could be expected when using more representative data, we find in our data a more balanced distribution of positive and negative experiences related to Internet use and fewer reports of problems related to Internet use.

The recent development within addiction studies to consider the inclusion of non-substance or behavioural addictions—e.g., Internet addiction—as formal diagnosis in a nomenclature such as the DSM 5 is a clear sign of an ongoing transformation of the conceptual framework for the underlying phenomena. Although Petry (p. 142, [27]) might be correct in her claims that behavioural addictions “share many features” with addictions related to the consumption of substances such as heroin or cocaine, it is nevertheless obvious that there is also a distinct difference between a substance addiction and a behavioural addiction, in the sense that the former category has been identified as addiction due to specific characteristics of a limited set of substances. In the beginning of the 1970’s Smith and Gay [28] published a book with the title “It’s so good do not even try it once—Heroin in perspective”. The price for the expansion of addiction to encompass various other non-substance-related behaviors is a considerable switch, possibly undermining or at least shifting attention away from the presumption that certain substances in and of themselves are capable of enslaving those who consume them due to inherent qualities of the substances. 

The distribution of hours per week on Internet at home in our study (Figure 1) demonstrates similarity with the well-known distribution of alcohol consumption, *i.e.*, the distribution is positively skewed with the tail consisting of individuals that have an extensive Internet involvement. This observation underlines that many of the studies that report alarming prevalence rates of extensive Internet involvement/addiction (see e.g., [16,17]) are likely to be huge overestimations of what proportions of Internet users that might considered to exhibit problematic use. It seems quite clear that this type of overestimation in the general case is a consequence of the fact that many studies has relied on convenience samples that are self-selected through advertisements on the Internet. While the respondents identified as “dependent” (80 percent of the total) in Young’s [16,17] study spent an average of 38.5 hours per week on the Internet “for pleasure”, the mean value for similar activity in the sample presented in this study was 10.7 hours per week and only 5 percent spent more than 30 hours per week online. 

As pointed out in the foregoing, it is of vital importance to reiterate that “Internet use” is a highly abstract and non-specific [23] category, and that it is by no means self-evident what functions different activities, made possible through the Internet, have for different types of individuals. Shen and Williams [6] identify two basic perspectives on the effects of Internet use. On the one hand there is a “compensation model” in which it is postulated that Internet use provides social options that are most beneficial for individuals who are less socially competent in “real life”. On the other hand there is also a model which suggests a polarization between resourceful and more disadvantaged individuals, *i.e.*, a model proposing that the “rich get richer” while at the same time the “poor get poorer.” Although the data in the present study does not allow for a more detailed analysis of these models, or other models, we can at least find some support for the presence of both positive and negative social effects. Regarding positive effects, a substantial number of individuals have had more social contacts with both family members as well as friends due to their use of Internet. Regarding negative effects, there is a decline in face-to-face contacts with both of these groups. However, taken together, there is a net sum increase in social contacts of 28% (given that we consider it possible to group the both categories of contacts together). 

About 50% of the respondents have indicated some experience of at least one problem related to their use of the Internet, but only 2.5% have marked the presence of all five problem items. In the regression analysis based on the summed occurrence of problem indicators there are three significant predictors that, besides weekly hours on Internet, relate to “time”. Gaming and membership in online communities are both time consuming activities. The significant effect of gambling, despite its relative low prevalence in the sample, points in the direction of a different type of relation to the dependent variable than those of gaming and membership in online communities, *i.e.*, gambling is closer to being a problem in itself. 

In possibly the most well developed field of addiction studies, that of alcohol addiction, the main general limitation in the “epidemiological approach”—that we have relied on in this study—is known to be the relative difficulty of reaching individuals with a high level of problems through the distribution of a survey. It is reasonable to expect that a similar self-selection effect is present in studies on problems related to Internet use, but it is not self-evident that the magnitude of such a problem will be the same as in alcohol studies. It is general knowledge that the social stigma associated with alcohol addiction is not present in the same way for individuals with problems related to extensive Internet use. Hence, they could be expected to be more inclined to participate in surveys (compared to individuals with alcohol problems). At the same time the classical divergence between “treatment seeking populations and larger realities” in the alcohol field [29] is not present when it comes to problems related to Internet use. This is due to the fact that a majority of the selected samples that have been used to illuminate problematic Internet use does not consist of treatment seeking individuals. Instead recruitment is often done through web-searches related to Internet use. Even though the epidemiological approach is associated with some methodological problems, it nevertheless seems to be worthwhile to continue to study the distribution of internet use and problems in the general population, and thereby provide a wider perspective on problems related to Internet use.

A fundamental component of most perspectives on the addiction phenomena is that addiction is persistent, *i.e.*, it is characterised by the fact that it has a strong tendency to not go away. This aspect of the addiction phenomenon cannot be addressed with reference to the empirical material presented in the present paper. However in the study by Van Roij *et al*. [20], a design with repeated cross-sectional surveys opened up for the identification of longitudinal cohort of online adolescent gamers (n = 467). Although the time frame for this group covered just one year (from 2008 to 2009) only half of the respondents that were classified as addicted in 2008 could be identified in the same category in 2009. This finding implies that extensive online gaming may be characterized as transitory rather than persistent, and hence does not have a clear connection to one of the most central characteristics of a traditional understanding of addiction. On the other hand, other addictions do tend to be more transitory among adolescents than adults. Thus, that study may not be applicable to the current sample, or to addiction phenomena among adults.

One limitation in the present study is that we attempt to contrast our results with Young’s although we do not have data for a “full” replication of the Young study. In the survey we used for analyses only five of the eight indicators used by Young were included. We would of course have preferred to have employed all eight indicators to work with. Due to survey space limitations, this was not possible. Another limitation is embedded in the cross-sectional design of the survey. Hence, we do not know how users of Internet develop extensive preferences or problems. Nor do we know the time order of predictors and dependent variables. There is much to be done in future studies using general population samples.
